# Natural Mineral Waters

**Published:** 1908-09-12

**Authors:** 


### THERAPEUTICS.

### NATURAL MINERAL WATERS.

The reason why natural mineral waters are so frequently prescribed instead of ordinary saline mixtures such as are found in the Pharmacopœias of all our hospitals (under the names of mistura alba, mistura magnesiæ sulph et carb., etc.), cannot be quite satisfactorily determined. Probably it is largely a matter of sentiment on the part of the general public. Those who can afford it go abroad for a "cure"; those who cannot, like at least to feel that they are drinking of the same healing spring as their wealthier neighbours. It is, of course, possible that radio-activity or some such obscure factor may enhance the therapeutic value of the freshly drawn water of a mineral spring, but this would hardly influence the bottled and exported article. But on the whole the fancy is a harmless one, and if the general public prefer a mineral water to a prescription, it is quite certain that they will get it; the doctor should therefore see that the mineral water chosen is a genuine and efficacious remedy, that it is taken in the proper manner, and only by those to whom it is suited.

*Hunyadi János* contains in one pint about 140 grains each of the sulphates of magnesium and sodium, together with 12 grains of sodium chloride. Other estimates give a higher percentage of the two first constituents. It thus ranks among the more powerfully aperient salines, while the amount of magnesium sulphate gives it a characteristic bitter taste. The ordinary dose—about four ounces—contains roughly 30 grains of each active constituent. The effect of the sodium chloride is mainly to mitigate the bitter taste of the sulphates.

The water should be taken with or without the addition of a small tumbler of hot water first thing on rising in the morning. Hot water aids the purgative action. Some patients find it more efficacious when sipped during the operation of dressing, others prefer to take it at a draught. In some cases it may be prescribed with twice as much Carlsbad water, made warm—*i.e.*, one wineglassful of Hunyadi and two wineglassfuls of Carlsbad. The administration should be occasional, and should not be continued every morning for more than a fortnight. Usually the dose is ordered two or three times a week until the action of the bowels becomes regular. In some cases it is necessary to prescribe a pill of aloin or a dose of cascara the evening before.

The main action is to prevent the absorption of water from the upper bowel, the contents of which remain fluid and bulky, and pass rapidly and easily into the lower portions of the intestine. The sulphates are hardly absorbed at all; the small amounts which pass into the blood stream probably have no further effect than to increase diuresis during their excretion by the kidneys.

Hunyadi János is not suited for continuous administration. It should be given occasionally, either to meet the needs of an occasional constipation, or in chronic cases when special indications arise. Thus at the beginning of a course of treatment a week or so of Hunyadi János may be a useful adjunct to dietetic and other measures, or later on when the routine prescribed is not enough to maintain the daily action, it may be ordered two or three times a week till the condition is improved. The special conditions for which Hunyadi János is suited are chronic catarrhal inflammation of the bowels, and the gouty or "bilious" diatheses. The sulphates contained in the water do not irritate the mucous membrane, while acting very effectively in removing any excess of mucus; their diuretic action is an additional recommendation in gout. It has not been shown that they exercise any special influence on the liver, but they are certainly useful in the disorders popularly ascribed to that organ, mainly because they deplete the abdominal vessels, and relieve the passive hyperæmia of the splanchnic and portal systems, upon which probably much of the feeling of "fullness" and discomfort of certain dyspeptics depends. Hunyadi János is not suitable for anæmic patients, perhaps owing to the altered osmotic conditions of the blood in that disease. Convalescents and those suffering from chronic ulceration of the bowels are also unfit subjects for this remedy. It has no action on gastric ulcers, and, in fact, its effect on the stomach altogether is practically nil. After a time a certain amount of habituation takes place, and this is one reason why it should not be employed continuously for more than a short time. The bitter taste of Hunyadi renders it an unsuitable aperient for children.

